# 

**Published:** 1892-05-21

**Authors:** 


The Doctor's Armchair.—Secret Remedies . . . 115

The Attitude of Great Writers towards Disease.—II. Shakespeare . . . . 116

Medical History from the Earliest Times. By E. T. Withington, M.A., M.B.Oxon. IX.—The Philosophers—Democedes—The Æsclepidæ . . . . 117

Everybody's Page . . . . . 118

Annotations.—Curiosities of Medical Politics—Why will Doctors not Save?—English Ballet-Girls Abroad . . . 119

Notes and News . . . . . 120

Around the Hospitals—Scraps and Gleanings . . . 121

The Practitioner's Mirror and Retrospect—

Medicine.—Elongated Uvula—Salicylic Acid in the Treatment of Acute Rheumatism . . . . 122

Ophthalmology.—Treatment of Trachoma . . . . 124

The Practitioners' Bookshelf.—A Medical Year Book . 124

The Institutional Workshop—

Within the Hospitals.—II.—Royal National Hospital for Consumption, Ventnor, 125; Alteration at Rotherhithe Infirmary 127

Festival Dinners.—I.—King's College Hospital, 127; II.—The Orphan Working School . . . . 128

The Student of Medicine.—Appointments—Vacancies.

### EXTRA SUPPLEMENT.—THE NURSING MIRROR.

En Passant.—The Nurses' Co-operation—The London Temperance Hospital—A Diet Dispensary — Dr. Sutton and his Students—The Faddists of the Day—The Insane of Montenegro—The Proposed Nursing Institution at Birmingham—Northern Workhouse Nursing Association—Short Items . liii

Ventilation, Disinfection, and Diet. By P. Caldwell Smith, M.D. VI.—Disinfection . . . . liv

Nursing in Workhouse Sick Wards . . . . lv

Rural District Nursing and the Jubilee Institute . lv

For Reading to the Sick.—Heavenly Love . . . lv

Among the Institutions—Where to Go . . . . lvi

Everybody's Opinion.—Cork and its Neighbourhood—The Lowestoft Hospital . . . . lvi

Experiences of a Trained Nurse in Valparaiso During the Revolution (concluded) . . . . lvii

Notes and Queries . . . . lvii

Matrons . . . . . lviii

Technical Teachers . . . . lviii

Four Months in a Hospital Ward (continued) . . . lix

Off Duty.—Tired of Life . . . . . lx

